# Pulmonary rehabilitation efficacy on quality of life and functional capacity in interstitial lung disease

**DOI:** 10.6026/973206300212747

**Published:** 2025-08-31

**Authors:** Mohammed H. Albitar, Gayathri G., Adithya Andanappa, Hanisha Reddy Kukunoor, Gyanendra KC., Anusha Garapati, Asiya Tasleema Shaik, Freeha Rana, Mohamed Ali, Mohammed Abdul Mateen

**Affiliations:** 1 Department of Internal Medicine, College of Medicine, Alfaisal University, Riyadh, Saudi Arabia; 2 Department of Surgery, Karnataka Institute of Medical Sciences, Hubbali, India; 3 Department of Internal Medicine, Mysore Medical College and Research Institute, Mysore, India; 4 Department of Internal Medicine, Maheshwara Medical College and Hospital, Hyderabad, India; 5 Department of Population Health, UNSW School of Medicine & Health, Sydney, Australia; 6 Department of Internal Medicine, College of medical sciences, Bharatpur, Nepal, India; 7 Department of Internal Medicine, Gandhi Medical College, Hyderabad, India; 8 Department of Internal Medicine, Ross University School of Medicine, Bridgetown, Barbados; 9 Department of Internal Medicine, Victoria Hospital, Kirkcaldy, United Kingdom; 10 Department of Internal Medicine, Shadan Institute of Medical Sciences, Hyderabad, India

**Keywords:** Compliance, Functional capacity, Health-related quality of life (HRQOL), interstitial lung disease (ILD), pulmonary rehabilitation (PR)

## Abstract

The impact of pulmonary rehabilitation (PR) on health-related quality of life (HRQOL), functional capacity and compliance in patients with interstitial lung disease (ILD) is of interest. Ten patients participated in a 3-month PR program, which significantly improved 6-minute walk test (6MWT) distance and St. George's Respiratory Questionnaire (SGRQ) scores. High compliance (90%) was observed, with improvements in breathlessness and recovery perception. Thus, we show that PR is effective in enhancing HRQOL and functional capacity in ILD patients. Larger studies are needed to confirm these findings and assess long-term benefits.

## Background:

Interstitial lung disease (ILD) refers to a group of disorders marked by progressive pulmonary fibrosis, leading to exertional dyspnea, hypoxemia and systemic deconditioning [1]. These manifestations significantly impair patients' physical function, psychological well-being and overall quality of life. Among ILD subtypes, idiopathic pulmonary fibrosis (IPF) is the most prevalent and serious condition, presenting as a chronic and progressive fibrotic lung disease of unknown etiology. IPF incidence is estimated at 3-9 cases per 100,000 individuals in North America and Europe [2]. Despite advances in pharmacological treatments, the median survival after IPF diagnosis remains low, typically ranging from 2 to 5 years. Progressive dyspnea, exercise intolerance and skeletal muscle dysfunction further limit daily functioning and diminish health-related quality of life (HRQOL) [3]. Pulmonary rehabilitation (PR) is a multidisciplinary, non-pharmacological intervention combining structured exercise training, education and psychological support and is widely established for managing chronic obstructive pulmonary disease (COPD). Its efficacy in improving exercise capacity, alleviating dyspnea and enhancing HRQOL in COPD patients has prompted its use in other chronic lung diseases such as ILD [4, 5]. However, ILD has unique pathophysiological characteristics, including a greater propensity for exercise-induced hypoxemia, pulmonary hypertension and ventilatory limitations, which may influence the outcome of PR. These differences necessitate more detailed evaluation of its role in ILD management. Patient-reported outcome measures (PROMs) have emerged as an indispensable tool for evaluating the effectiveness of PR interventions [6]. They offer direct insights into health status, functional ability, psychological state and overall quality of life [7]. In ILD, PROMs provide a comprehensive evaluation of the disease's physical, emotional and social impact beyond conventional clinical parameters. Among these measures are assessments of the severity of dyspnea, levels of fatigue experienced, emotional well-being, and functional limitations. Validation of PROMs in ILD offers key evidence that interventions such as PR effectively reduce symptoms, increase functional capacity and promote improved HRQOL [8].

While pulmonary rehabilitation holds potential for improving ILD-related symptoms, most supporting data originate from research on COPD and similar obstructive pulmonary disorders [9]. Strong data on the specific benefits of PR in patients with ILD, particularly concerning PROMs, are scarce. Moreover, regional disparities, such as the limited availability of PR programs in resource-constrained settings, such as India, further underscore the need for localized studies [10]. The impact of PR on PROMs in ILD remains underexplored despite its potential to address key challenges such as exertional dyspnea, skeletal muscle dysfunction and reduced exercise capacity. This study aims to evaluate the efficacy of pulmonary rehabilitation in improving patient-reported outcome measures in patients with ILD [11]. By focusing on PROMs, this research seeks to assess the impact of PR on various dimensions of patient health and well-being, including the physical, emotional and social domains. This analysis offers valuable insights into the role of PR in improving quality of life in patients with ILD, supporting clinical guidelines and patient-centered care. Addressing some of these knowledge gaps, this study aims to contribute toward optimizing PR protocols and establishing their role in ILD management [12]. Therefore, it is of interest to effects of pulmonary rehabilitation on health-related quality of life and functional capacity in interstitial lung disease.

## Methodology:

This descriptive study was conducted at a tertiary respiratory care center in Hyderabad, India involving patients diagnosed with interstitial lung disease (ILD) following up in the outpatient setting. Data collection assessing the efficacy of pulmonary rehabilitation in ILD patients took place between 2023 and 2024 [13]. Participants were selected based on predefined inclusion and exclusion criteria. Inclusion criteria were: confirmed ILD diagnosis via high-resolution computed tomography (HRCT), histopathology, or multidisciplinary discussion; age above 30 years; abnormal pulmonary function tests abnormalities, defined as forced vital capacity (FVC) < 80% predicted or diffusion capacity of the lungs for carbon monoxide (DLCO) < 80% predicted; and a six-minute walk distance (6MWD) of ≥150 meters. Exclusion criteria included: right ventricular systolic pressure > 55 mmHg, advanced heart failure, extreme physical limitations (6MWD < 150 m or > 500 m), severe airflow obstruction (FEV1/FVC ratio < 0.7), pregnancy, or any clinical condition that could compromise mobility or participation in the study. Ten participants were enrolled, representing the centers expected patient throughput over a three-month period. Data collection included demographic and clinical details, spirometry in accordance with American Thoracic Society (ATS) guidelines and the St. George's Respiratory Questionnaire (SGRQ) for assessing HRQOL. Functional capacity was measured using the six-minute walk test (6MWT). Follow-up assessments were conducted three months after the intervention using the same tools to compare outcomes. The data were analyzed using SPSS version 22.0. Quantitative variables were reported as means with standard deviations; qualitative variables were expressed as percentages. Paired and unpaired t-tests were used to compare the pre- and post-intervention SGRQ scores, with a statistical significance defined as p < 0.05. Following baseline assessments, ten participants were enrolled in a structured three-month pulmonary rehabilitation program at the Department of Cardiopulmonary Rehabilitation. The intervention was initiated with two weeks of supervised sessions, held three times per week for two hours each, under the supervision of trained physiotherapists and pulmonologists to ensure safety, proper exercise techniques and understanding of the program. Following this initial phase, participants transitioned to a home-based program for ten weeks, with adherence monitored via weekly calls and bi-weekly clinic visits. All participants continued to receive optimal medical management alongside the rehabilitation program. After the intervention, participants were reassessed using the SGRQ, spirometry and 6MWT. Ethical approval was obtained from the Institutional Research Committee and written informed consent was secured from all participants prior to enrollment. Data confidentiality was maintained and participants retained the right to withdraw at any point during the study.

The pulmonary rehabilitation program was structured to address the complex needs of ILD patients through integrated physical training, education and psychological support. Exercise sessions included moderate-intensity aerobic activities (walking, treadmill use, cycling) and strength training of the upper and lower limbs using light weights, resistance bands, or body weight tailored to individual tolerance levels. Breathing techniques, including diaphragmatic and pursed-lip breathing, were used to reduce dyspnea and enhance oxygenation. The educational segment focused on disease understanding, treatment adherence, vaccination and hygiene practices, delivered through both individual and group counseling sessions. Psychological and emotional support addressed anxiety and depression using guided meditation and relaxation strategies. Nutritional counseling was offered when appropriate, along with smoking cessation support to reduce the risk of disease progression. Patient safety was continuously monitored throughout the program. Oxygen saturation was monitored during sessions and maintained at or above 90%. Heart rate and blood pressure were recorded before, during and after exercise to detect any adverse events. Participants were trained to recognize and report signs of clinical deterioration, including worsening dyspnea or chest pain. The primary outcome was improvement in quality of life, as indicated by changes in SGRQ scores. Secondary outcomes included improvement in functional capacity (6MWD) and pulmonary function (FVC and DLCO). This structured approach ensures patient-centered, holistic pulmonary rehabilitation by emphasizing physical conditioning, educational engagement and psychological recovery and aims to overcome personal barriers, improve adherence and enhance clinical outcomes, thereby improving the quality of life of patients with ILD.

## Results:

The study included 10 participants with an equal gender distribution: 50% male and 50% female. Educational levels were varied: 30% were illiterate, 40% had primary education, 20% completed high school and 10% held postgraduate degrees. Occupational exposure showed that 30% held high-risk jobs, while 70% worked in low-risk settings. The majorities (80%) of participants were married (Table 1 - see PDF). Idiopathic Interstitial Pneumonia (IIP) was the most common diagnosis, accounting for 70% of the cases. Among IIP cases, usual interstitial pneumonia (UIP) was present in four patients, while IPF-UIP and UIP-ILD were each seen in one. Combined Pulmonary Fibrosis and Emphysema (CPFE) accounted for 20% of the cases and Connective Tissue Disease-Associated ILD accounted for 10% (Table 2 - see PDF). 6-Minute Walk Test and SGRQ Scores: Paired t-tests demonstrated significant post-intervention improvements in both the St. George's Respiratory Questionnaire (SGRQ) and 6-Minute Walk Test (6MWT) scores. Paired t-tests were used to assess the changes in the symptom components of the SGRQ. (Table 3 - see PDF) presents the mean, standard deviation, standard error mean, t-values, and p-values for changes in the 6MWT distance, SGRQ weight, and total SGRQ score following the pulmonary rehabilitation program. Significant improvements were observed across all measures, with the 6MWT distance showing a mean increase of 47.2 meters (p = 0.001), indicating clinically meaningful improvement in exercise capacity. Both the SGRQ weight and total score also showed significant reductions, suggesting improvements in health-related quality of life (HRQOL) following rehabilitation (p = 0.001 for both measures). While most symptom pairs showed no statistically significant changes, Pair 5 ("Number of respiratory infections in the past year") showed a significant increase (mean difference = 9.90, p = 0.02) (Table 4 - see PDF). Analysis of the SGRQ impact domain revealed significant improvements. For instance, Pair 6 ("Getting breathless when talking") and Pair 14 ("Expectation of no improvement in chest problems") both showed statistically significant changes post-intervention (Table 5 - see PDF). Pearson's correlation analysis indicated a strong negative association between increases in 6MWT distance and decreases in SGRQ scores. Additionally, changes in SGRQ scores were strongly correlated with changes in its component scores (Table 6 - see PDF).

## Discussion:

This study evaluated the effectiveness of a structured three-month pulmonary rehabilitation program on HRQOL, exercise capacity and treatment compliance in patients with ILD. Significant improvements were observed across the key outcomes, highlighting the potential benefits of pulmonary rehabilitation in this patient population [14]. The improvement in the overall scores for the total SGRQ after pulmonary rehabilitation indicates a positive effect of the intervention on HRQOL. A statistically significant reduction in the SGRQ scores (mean difference = 17.24, p = 0.001) suggests improved well-being and reduced disease burden. These findings are consistent with previous studies that have shown that PR is effective in enhancing HRQOL in chronic respiratory diseases, such as COPD and ILD [15]. Although the overall SGRQ scores showed statistically significant improvement, individual component analysis revealed mixed outcomes. The symptom domain did not show a significant change, suggesting that while PR may improve the overall quality of life, its impact on perceived respiratory symptoms might be limited [16]. This may be due to the chronic and progressive nature of ILD, in which structural lung changes are not reversible even with rehabilitation. However, the activity and impact components demonstrated variable improvements, particularly in psychosocial aspects, which reflected better emotional well-being and participation in daily activities [17]. Sociodemographic factors, such as education, occupation and residence, did not significantly influence SGRQ scores, suggesting that the benefits of pulmonary rehabilitation may be uniformly accessible across diverse patient populations. These results highlight the need to address all HRQOL domains to achieve comprehensive patient-centered outcomes [18]. The significant improvement in the 6MWT distance (mean difference = 47.2 m, p = 0.001) highlights the efficacy of pulmonary rehabilitation in enhancing functional exercise capacity. This improvement exceeded the minimally important difference threshold (25-35 m), indicating clinically meaningful improvement. These findings align with those of previous studies that reported increased exercise tolerance following pulmonary rehabilitation in patients with ILD [19]. The mechanisms underlying these improvements include enhanced cardiovascular fitness, increased respiratory muscle strength, improved muscle conditioning and symptom management. However, the exact physiological adaptations remain unclear and further research is needed to identify the specific contributors to improved exercise performance. Increased exercise capacity has important implications for daily functioning, enabling patients to perform better physical activities and potentially improving their overall quality of life [20].

The study recorded a 90% compliance rate, with nine of ten patients completing the full program. This finding is consistent with previous studies reporting moderate to high adherence rates to PR in chronic lung conditions [21]. In a study by He *et al.* (2025) [22], the effectiveness of a structured three-month pulmonary rehabilitation (PR) program was evaluated in patients with interstitial lung disease (ILD), focusing on health-related quality of life (HRQOL), exercise capacity, and treatment compliance. The results highlighted significant improvements across key outcomes, particularly in HRQOL and functional capacity, emphasizing the potential benefits of PR in managing ILD. Addressing this issue requires adequate logistical support to improve accessibility. Barriers to adherence in this study included transportation difficulties, financial constraints, physical limitations and limited awareness of PR benefits. These findings align with the existing literature, which cites logistical and motivational factors as key contributors to nonadherence. Improving patient education and awareness, addressing financial and transportation barriers and providing individualized rehabilitation plans can improve compliance and optimize PR outcomes. The results of this study underscore the potential of pulmonary rehabilitation as an integral component of ILD management. Improvements in exercise capacity and HRQOL highlight their role in addressing the physical and psychosocial challenges associated with ILD. However, the variability in component-specific outcomes suggests the need for tailored interventions to maximize benefits across the HRQOL spectrum. Maximizing the benefits of pulmonary rehabilitation requires a multi-intervention approach, including strategies to overcome barriers, enhance patient education and tailor exercise plans to individuals' needs and limitations. This study had a small sample size, which limits the generalizability of these findings. Future studies with larger cohorts are needed to validate these findings and identify subgroups that may benefit the most from pulmonary rehabilitation. Additionally, this study did not consider long-term outcomes; therefore, further research is necessary to establish whether these benefits are sustained over time. Another limitation is the use of self-reported data on barriers and compliance, which may be susceptible to recall bias. Future studies should include objective adherence measures and explore alternative delivery models, such as home-based and telerehabilitation programs, to enhance accessibility.

## Conclusion:

A structured three-month pulmonary rehabilitation program significantly improved health-related quality of life and exercise capacity in ILD patients, with high adherence. While total SGRQ and 6MWT scores improved, variability in symptom domains suggests the need for tailored interventions. Enhancing education and overcoming logistical barriers is crucial for optimizing outcomes.
